# On the Character of Consciousness

**DOI:** 10.3389/fnsys.2016.00027

**Published:** 2016-03-30

**Authors:** Arto Annila

**Affiliations:** ^1^Department of Physics, University of HelsinkiHelsinki, Finland; ^2^Department of Biosciences, University of HelsinkiHelsinki, Finland

**Keywords:** causality, cognition, free energy, non-determinism, the principle of least action, the second law of thermodynamics

## Abstract

The human brain is a particularly demanding system to infer its nature from observations. Thus, there is on one hand plenty of room for theorizing and on the other hand a pressing need for a rigorous theory. We apply statistical mechanics of open systems to describe the brain as a hierarchical system in consuming free energy in least time. This holistic tenet accounts for cellular metabolism, neuronal signaling, cognitive processes all together, or any other process by a formal equation of motion that extends down to the ultimate precision of one quantum of action. According to this general thermodynamic theory cognitive processes are no different by their operational and organizational principle from other natural processes. Cognition too will emerge and evolve along path-dependent and non-determinate trajectories by consuming free energy in least time to attain thermodynamic balance within the nervous system itself and with its surrounding systems. Specifically, consciousness can be ascribed to a natural process that integrates various neural networks for coherent consumption of free energy, i.e., for meaningful deeds. The whole hierarchy of integrated systems can be formally summed up to thermodynamic entropy. The holistic tenet provides insight to the character of consciousness also by acknowledging awareness in other systems at other levels of nature's hierarchy.

## Introduction

Cognition is an ability that one has inherited from the evolutionary course of human species and its ancestors as well as accumulated in the course of one's own life from numerous experiences and incidences during diverse developmental and maturation processes. To perceive cognition in this way as a product of various processes raises a profound question: What is a change? Namely, an event, development or evolution as a whole ultimately consists of changes from one state to another. The decimation of any process to a series is familiar from physics but the conceptualization is not remote to neuroscience either (Fingelkurts and Fingelkurts, 2001; John, 2002; Perlovsky and Kozma, 2007; Freeman and Vitiello, 2009; Fingelkurts et al., 2010a, 2013). Moreover, cognition is not only one's arsenal from the past, but a present process for one to target toward future. Consequently we think that the concept of change is pivotal in comprehending cognition in general and its consciousness character in particular.

We are further motivated to make sense of cognition using the universal notion of change because the human brain, as the primary premise of cognition, displays in its structures and functions the same patterns as numerous other systems throughout nature (Linkenkaer-Hansen et al., 2001; Eguíluz et al., 2005; Mäkelä and Annila, 2010; He et al., 2013). For example, neural activity is no different from seismic activity, both comply with power laws (Touboul and Destexhe, 2010). A neuronal network, just as the World Wide Web, has a skewed distribution of nodes' degrees (van den Heuvel et al., 2008). Neural activity exhibits waves, oscillations, spiraling sequences and at times chaotic behavior just like economic activity displays cycles, trends and occasionally tumultuous conducts (Schroeder, 1991; Huang et al., 2010; Friedman and Landsberg, 2013). No question, the ubiquitous patterns have been recognized in diverse disciplines including neuroscience (Chialvo, 2010), but the main point remains unappreciated: The common characteristics result from natural processes, that is, from series of changes.

Evolution of any kind, when broken down to a succession of changes, can be given by an equation of motion. In this way the thermodynamic theory explains the recurrent patterns to result from least-time free energy consumption (Sharma and Annila, 2007). In other words, the skewed distributions are energetically optimal, and hence their cumulative sigmoid growth and decline curves are also optimal in energetic terms. The power laws, in turn, are ubiquitous by being central approximations of the sigmoid curves. The evolutionary equation asserts that natural systems evolve in non-deterministic and path-dependent manner (Annila and Salthe, 2010a). Also cognitive processes, unmistakably learning and decision making, share these universal attributes (Arthur, 1994; Bassanini and Dosi, 2001; Anttila and Annila, 2011). For these reasons we are motivated to employ the general theory to make sense of cognition and especially of its seemingly elusive conscious character.

Disciplines have branched far from their common stem in natural philosophy, and hence holism is today an unconventional tenet. Thus, our assertion that the human brain is no different by its operational and organizational principle from any other system in nature may appear odd and groundless at first sight. To justify our reasoning we will begin by outlining the thermodynamic theory (Chapter 2) and thereafter work insight to consciousness by relating the holistic perspective to various puzzles, phenomena, and well-known stances (Chapter 3). Finally, we summarize conclusions of the thermodynamic tenet to further debate and discourse (Chapter 4). As it will become apparent, our study does not yield groundbreaking resolutions, rather it substantiates common sense by a firm formalism.

## Thermodynamics of open systems

We reason that the human brain is no different from other systems in nature because its structures and functions display the ubiquitous patterns, i.e., distributions that sum up along sigmoid curves which, in turn, mostly follow power laws. Hence, the brain ought to be described and comprehended in the same way as any other system.

To this end the general principle of nature is known, in fact by many names, most notably as the second law of thermodynamics, the principle of least action and Newton's second law of motion. These three laws appear as if they were distinct from one and other when erroneously expressed in their determinate, i.e., calculable forms. For example, textbooks tend to present Newton's second law of motion so that force F = *m*a equals mass *m* times acceleration a = *d*_*t*_v, i.e., the change in velocity v. However, Newton himself wrote that the force F = *d*_*t*_p equals a change in momentum p, which yields by the definition p = *m*v not one but two terms F = *md*_*t*_v + v*d*_*t*_*m*. The change in mass relates via *dm* = *dE*/*c*^2^ to dissipation of photons ultimately to the cold space. Dissipation is inherent in any change, and hence it is also integral to cognition.

Likewise, the principle of least action in its original form due to Maupertuis includes dissipation in contrast to the familiar constant-energy, hence deterministic Lagrangian (De Maupertuis, 1746; Tuisku et al., 2009). Furthermore, statistical mechanics, as the probabilistic many-body theory underlying thermodynamics, can be formulated for open dissipative systems. However, when imposing the constant-energy condition, statistical mechanics limits to stationary systems (Kondepudi and Prigogine, 1998).

Dissipation, despite being an integral component of any change, may still appear as a downright secondary byproduct of neural activity. Yet, when a systems theory misses even a single and seemingly insignificant photon, such a theory does obviously not account for everything and leaves room for unaccounted effects, surmise and speculation. Of course, when probing neural activity in practice, knowledge of numerous factors will remain imperfect, but all the more the theory's bookkeeping of causes and effects, i.e., forces and ensuing motions, ought to be perfect.

### The physical basis

Today, when complex systems are more often modeled and simulated than described and explained, our ambition to account for everything with accuracy and precision extending down to a single photon might seem as an exceptional, perhaps even as an unattainable and abstract attempt. Therefore, it is worth stressing that for us an explanation is genuine only when it relates to everyday experience. For instance, the well-known conjecture that quantum mechanics could underlie consciousness (Bohm, 2002; Pylkkänen, 2014) does not qualify for us as an explanation, because entangled and superposed states do not make sense to us. The legendary illustration of a microscopic system being in two states at the same time by a cat being alive and dead at the same time simply does not seem sensible to us. The observed indeterminism implies to us that we just do not know the state of cat that goes missing. Likewise, we refute the idea in statistical mechanics that an observable state would sum up from a probability distribution of microscopic configurations, because the microstate (Mandl, 1971), in contrast to the state, is a theoretical concept without a discernable counterpart. In practice one microstate cannot be distinguished from another.

Surely, our stance can be argued against by claiming that not everything is necessarily tangible to the human being, but then again no observation is either free from some interpretation. Mere numbers mean nothing. Thus, mere agreement with recordings is no guarantee that non-determinism and purported non-localism as well as emergence could not be explained without conceptual conundrums (Annila and Kallio-Tamminen, 2012). It is worth noting that Schrödinger equation is devoid of dissipation (Griffiths, 2004), and hence it does not comply with observations that all changes are dissipative. Likewise, the textbook statistical mechanics accounts for the system when at thermodynamic equilibrium, not when in dissipative evolution from one state to another (Gibbs, 1902).

We think that the theory of cognition ought to be given in the form of an equation because mathematical notation leaves less room for ambiguity than natural language. By the same token, Darwin's theory, as the corner stone of biology, is not a theory by standards of physics but a narrative, albeit a conceivable one. Then again, an equation alone is no theory. Namely, when variables of a mathematical model fail to correspond to causes and effects, there is no enlightenment.

Traditionally rules and regularities have been deduced from meticulous measurements. Kepler's laws are examples of formalized observations. In neuroscience this approach is hardly an option. Recordings do not reproduce precisely enough to infer an equation of motion. Instead mathematical models, such as Markov chains that mimic data, more or less, are fashionable in providing predictions, at least trends (Laing and Lord, 2009). However, the model parameters do not map one-to-one with causes and effects. The introduced statistical indeterminism, i.e., randomness without reason, is not a substitute for non-determinism. It follows from the path-dependence of natural processes.

To obtain an equation by starting from an axiom is yet another possibility. For example, the axiom that inertia is distinguishable from gravity, known as equivalence principle, underlies general relativity (Misner et al., 1973). In neuroscience this approach for finding axioms does not appear amenable either. Recordings hardly display invariants to get hold of the foundation. Nonetheless, one may construct the theory by inferring or postulating self-evident axioms and challenge only ensuing conclusions (Tononi, 2008; Tononi and Koch, 2015). However, we would prefer axioms that are directly verifiable in terms of physics, but then neuroscience cannot stand out as a distinct discipline, its concepts cannot be chosen self-sufficiently, and its objects of study cannot be singled out as unique phenomena.

We find the ancient atomism (Berryman, 2011) as a sound and solid stance. It claims that everything comprises indivisible basic building blocks. Since the atom, as a chemical element, turned out to be divisible, the most elementary constituent was renamed as the quantum of action. The quantum of light is its most familiar embodiment. The human eye can register even a single photon and our skin is sensitive to photon influxes and effluxes that are sensed as hot and cold. Thus, the photons are real by everyday experience, and hence the quantum of action qualifies for us as a tangible entity. The quantum-embodied atomism is further motivated because every chemical reaction will either emit or absorb at least one photon. Also annihilation of matter with antimatter yields only photons. Also other observations substantiate the axiom that everything, and hence also cognition, is ultimately embodied by the quantized actions (Annila, 2010, 2012; Varpula et al., 2013). The atomism is not new to neurosciences either. It has been formulated in at least in neurophysiological context (Fingelkurts et al., 2009, 2010a).

The quantum of action has energy *E* and time *t* as its attributes, or equivalently momentum p and wavelength x, so that their product is invariant known as Planck's constant

(1) $$\begin{array}{r} h=Et=\text{p}\cdot \text{x}. \end{array}$$

In other words, energy and time do not exist as such. They are characteristics of the quanta (Annila, 2016). Surely, Equation (1) is mathematically equivalent to the textbook form *E* = *hf*, where frequency *f* = 1/*t*, but then it is not evident that *h* is the quantum's measure. The invariance means, for example, that the wavelength will change along with changing momentum but the photon itself remains intact. Consequently, we find virtual photons as an abstract theoretical construct without correspondence to reality (Peskin and Schroeder, 1995).

A system changes from one state to another by acquiring quanta from its surroundings or by losing quanta to its surroundings. Thus, the change in energy is, according to Equation (1), invariably accompanied with the change of time. This is common sense. For example, a chemical reaction will progress in the course of time by acquiring or expelling quanta that carry energy as heat until a stationary state has been attained. Many a biological system is recurrently subject to changes due to its changing surroundings. Therefore, animate will hardly ever attain and reside in thermodynamic steady states. Specifically, the central nervous system is incessantly receiving and sending impulses to its surroundings comprising the body and beyond.

In practice there is hardly a way to keep track of all quanta embodying even a microscopic system, but formally the system can be described with the precision of one quantum. This is not only a remarkable but consequential resolution. Not only is the neural network no different from any other energy transduction system, but the atomistic axiom excludes other factors. Put differently, if one were to argue that consciousness is not embodied by quanta, the stance would violate causality by introducing some other constituents from nothing. That is to say, a cause of any kind is ultimately nothing but an energy difference, i.e., some form of free energy. Its ensuing effect is nothing but a quantized flow of energy. Thus, causal power, as a characteristic of consciousness (Kim, 1992), is inherent in the thermodynamic description.

Our approach to account for the entirety in terms of quanta undoubtedly resembles reductionism. The idea that the system is nothing but the sum of its parts has been refuted, for instance, by referring to emergent characteristics of consciousness. Likewise, properties of a molecule cannot be inferred from properties of its constituent atoms. However, the molecule does not form only from atoms, but also from the photons that couple from surroundings to the synthesis (Pernu and Annila, 2012). If these quanta are not included in the description, obviously the molecular characteristics remain unaccounted. Conversely, no new property will appear from mere permutations of systemic constituents. Instead a novel characteristic will appear along with the flux of quanta from the surroundings to the system or *vice versa*. In other words, the monistic account (Stoljar, 2015) is in fact complete when every quantum of action is included. This essential role of surroundings in emergence has been pointed also in neuroscience (Rudrauf et al., 2003; Revonsuo, 2006; Fingelkurts et al., 2010b).

We realize that our physicalism does not immediately enlighten, for instance, subjective conscious experience, i.e., qualia, which is the contested concept about *the ways things seem to us* (Dennett, 1988; Chalmers, 1995). True enough, one does adhere meanings beyond mere perception. For instance, the sensation of red color is not only about registering corresponding energy of the photons at the retina, but the influx will trigger processes that involve more. What exactly is implicated may not be easily exposed in practice, but in any case we maintain that the supervening processes can be formally described with the exactness of one quantum.

### The systems description

**Figure 1:**
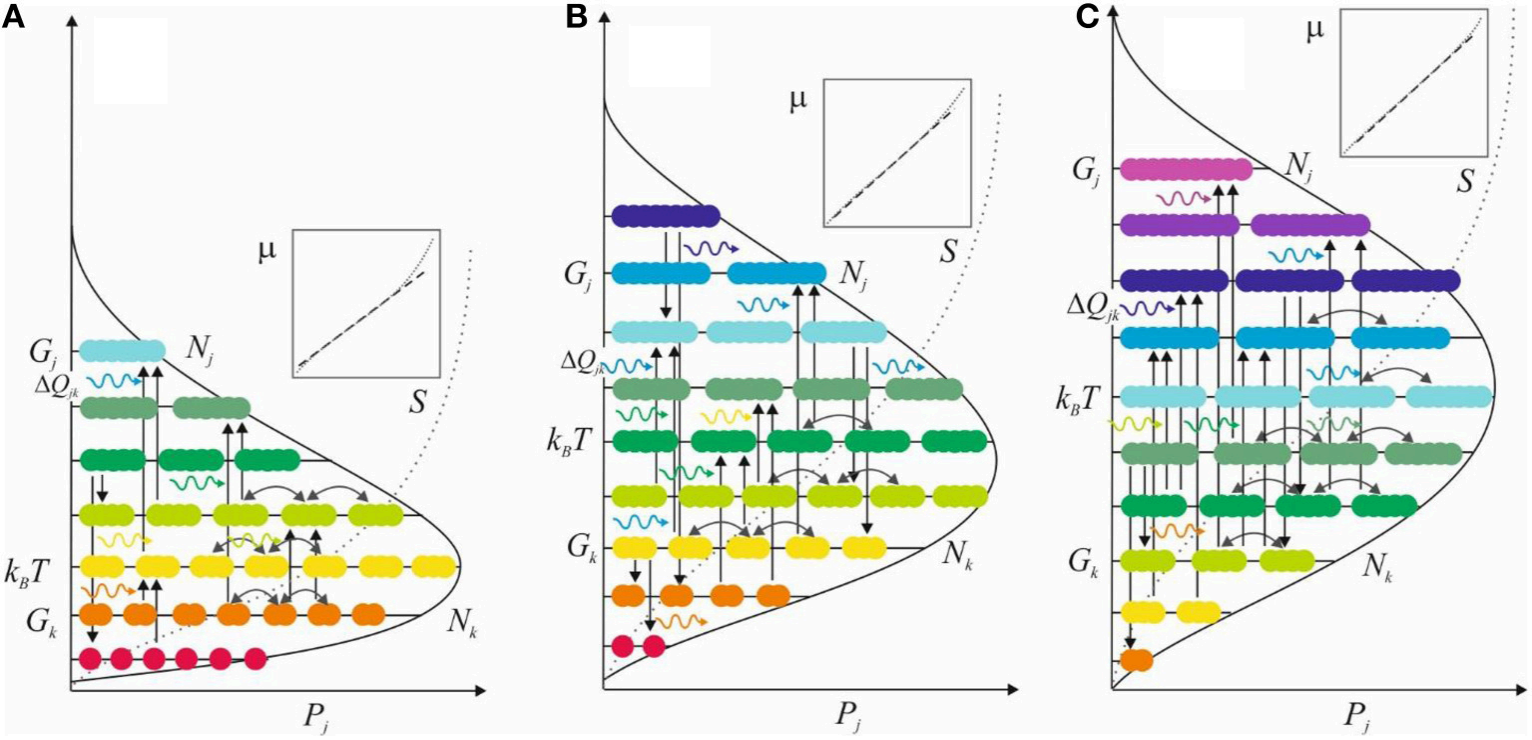
**System is portrayed in terms of an energy level diagram along its evolutionary path at three states (A–C)**. Each diagram pictures various populations *N*_*k*_ of entities, each with energy attribute *G*_*k*_. Vertical arrows indicate paths of transformations, i.e., changes from *k*-entities in the population *N*_*k*_ to *j*-entities in the population *N*_*j*_. Horizontal wavy arrows denote influx and efflux of photons that invariably couple to these transformations. Horizontal bow arrows, in turn, mean inconsequential exchange of indistinguishable entities. The system evolves, step-by-step, via absorptive and emissive *jk*-transformations from one state to another toward ever more probable partitions, denoted by *P* = ∏*P*_*j*_, eventually arriving at a stationary-state balance where its average energy *k*_*B*_*T* equals energy density in the system's surroundings. The outlined skewed partition accumulates along a sigmoid curve (dotted) which follows mostly a straight line on a log-log scale (insert) for entropy *S* = *k*_*B*_ln*P* vs. [chemical] potential energy μ.

The above preliminaries pave the way for the formal description of a system. Since all entities are understood to comprise of the basic building blocks, any entity can be related to any other in energetic terms. So, all those entities that one chooses to refer to as the system can be placed on an energy level diagram (Figure 1). This description can be formalized mathematically irrespective of complexity (Mäkelä and Annila, 2010).

According to the general theory of many-body systems the state can be expressed concisely and completely in terms of probability *P*. It is the measure of what it takes to have, for example, a pool of certain neurotransmitter molecules in a synaptic vesicle. Undoubtedly, it will take a lot of things. Precursors are needed for syntheses of transmitters as well as energy-rich chemicals are required to power the production. Moreover, machinery for the syntheses and molecular transport is necessary. In practice we do not know all factors that are involved in attaining the particular state of synaptic vesicle. Nonetheless, we may formally denote the probability *P*_*j*_ for the pool of neurotransmitters, in numbers *N*_*j*_, by accounting for all those vital ingredients, each in numbers *N*_*k*_, using the product form P_*k*_. It ensures that if any one of the vital ingredients is missing altogether, not a single neurotransmitter molecule will be found in the vesicle. Of course, it is not the mere number *N*_*k*_ of substrates that matters but also the substrate's energy attribute *G*_*k*_. Specifically, *P*_*j*_ depends on the difference between energy *N*_*k*_exp(*G*_*k*_/*k*_*B*_*T*) that is bound in the substrates and energy that is bound in the product exp(*G*_*j*_/*k*_*B*_*T*) as well as on the difference in energy that couples from surroundings via flux of photons to the synthesis of the *j*-entities from *k*-entities, i.e., exp(Δ*Q*_*jk*_/*k*_*B*_*T*). Formally this dependence of *P*_*j*_ on energetics is given by

(2) $$\begin{array}{r} P_{j}={\left[\underset{k=1}{\prod }N_{k}{\text{e}}^{-\Delta G_{jk}∕k_{B}T}{\text{e}}^{+i\Delta Q_{jk}∕k_{B}T}\right]}^{N_{j}}∕N_{j}! \end{array}$$

for the population of *N*_*j*_ products. All energy terms are relative to the average energy of the system per particle, denoted by *k*_*B*_*T* for historical reasons. The division by factorial *N*_*j*_! takes into account energetically equivalent permutations. It is worth emphasizing that these configurations, that the system cannot distinguish energetically, populate the same state. This is of course common sense. If one cannot distinguish one entity from another, one claims that they are identical. One's ability or any other system's ability to make a distinction requires ultimately recognition of some difference in energy. For the sake of clarity, imaginary part *i* in Equation (2) distinguishes energy in radiation, known as the vector potential, from quantized material forms of energy, known as the scalar potential (Figure 1). The probability of any other population can be denoted in the same way as *P*_*j*_. Then the total probability *P* of the whole system is simply the product

(3) $$\begin{array}{l} P=\underset{j=1}{\prod }P_{j}. \end{array}$$

Although the status of a system is accurately and precisely given by the product form (Equation 3), one would prefer an additive measure to make comparisons. In statistical mechanics entropy *S* is the additive measure of a system's status. It is obtained from the logarithm of *P*

(4) $$\begin{array}{r} S=k_{B}\ln\;P=\frac{1}{T}\underset{j=1}{\sum }N_{j}\left[k_{B}T+\underset{k=1}{\sum }\left(\mu_{k}-\mu_{j}+i\Delta Q_{jk}\right)\right] \end{array}$$

by multiplying with *k*_*B*_ for historical reasons. The shorthand notation μ_*k*_ = *k*_*B*_*T*ln*N*_*k*_ + *G*_*k*_, known as chemical potential for the logarithm of density in energy *N*_*k*_exp(*G*_*k*_/*k*_*B*_*T*) is expedient (Gibbs, 1902). Also Stirling's approximation ln*N*_*j*_! ≈ *N*_*j*_ln*N*_*j*_ – *N*_*j*_ is convenient. It holds the better the larger *N*_*j*_. For instance, when *N*_*j*_ > 100, the error relative to ln*N*_*j*_! < 1%. In the Equation 4 the first term sums all energy that is bound in the system's entities, including, for example, the pool of neurotransmitter molecules. The second term sums all energy differences, i.e., free energy terms that reside within the system as well as between the system and its surrounding systems, including, for instance, differences in electrochemical potentials across the vesicle's membrane. All these forces drive the system and its surroundings toward thermodynamic balance. The resulting change in entropy is obtained from the time differential

(5) $$\begin{array}{l} \frac{dS}{dt}=\frac{1}{T}\underset{j=1}{\sum }\frac{dS}{dN_{j}}\frac{dN_{j}}{dt}=\frac{1}{T}\underset{j=1}{\sum }\frac{dN_{j}}{dt}\underset{k=1}{\sum }\left(\mu_{k}-\mu_{j}+i\Delta Q_{jk}\right) \end{array}$$

Where *dN*_*j*_/*dt* denotes the change in *N*_*j*_ that result from consumption of free energy Σμ_*j*__*k*_ – μ_*j*_ + *i*Δ*Q*_*jk*_. For example, the accumulation rate of transmitter molecules in the synaptic vesicle

(6) $$\begin{array}{l} \frac{dN_{j}}{dt}=\frac{1}{k_{B}T}\underset{k=1}{\sum }\sigma_{jk}\left(\mu_{k}-\mu_{j}+i\Delta Q_{jk}\right) \end{array}$$

is proportional to free energy by rate parameters σ_*jk*_ > 0. Each parameter is associated with a transformation mechanism, such as an enzyme, that consumes free energy in the form of chemical energy. An energy transducer of any kind is according to the scale-free theory a system of its own. Hence, it is subject to changes too. For example, a mutation in a gene may lead to an altered catalytic activity, and hence affecting the flow rate from the substrates to the products and *vice versa*.

Although we do not know the details of how the system evolves from one state to another, the formal scale-free expressions (Equations 1–6) include every detail down to the precision of one quantum. In other words, the numerous flows of energy in a complex system are all formally included in Equation (5). Since there is no option to create the quanta from nothing or to destroy the quanta for nothing, the flows will have to direct along the least-time paths of free energy consumption. In biological terms evolution from one state to another will naturally select those means and mechanisms that facilitate survival. The scale-free patterns are consequences of this least-time imperative (Mäkelä and Annila, 2010).

When inserting Equation (6) to Equation (5), the quadratic form proves the second law of thermodynamics, i.e., *dS* ≥ 0 both for Σμ_*k*_ – μ_*j*_ + *i*Δ*Q*_*jk*_ > 0 and < 0. The thermodynamic entropy cannot ever decrease. For example, the neurotransmitter population will increase *dN*_*j*_ > 0 when there are resources Σμ_*k*_ – μ_*j*_ + *i*Δ*Q*_*jk*_ > 0 for its production. Conversely, the population will decline *dN*_*j*_ < 0 when Σμ_*k*_ – μ_*j*_ + *i*Δ*Q*_*jk*_ < 0. Thus, their product in Equation (5) is always non-negative.

It is worth stressing that entropy by Equation (4) is a measure of bound and free energy, not of disorder or the number of microstates. Although the definition of *P* by Equation (3) differs from the one referred to by the free-energy principle (Nicolis and Prigogine, 1977; Haken, 1983; Friston et al., 2006; Friston, 2010), the idea is the same: Evolution of any kind directs toward a free energy minimum state. The least-time principle parallels also of the principle of least effort (Zipf, 1949).

According to the holistic tenet any system is at the mercy of its surroundings. Therefore, changes in surroundings will manifest themselves as activity that will move the system in quest of regaining balance. For instance, when a neuron reverts its polarity, the synaptic vesicle will respond by releasing neurotransmitters. Conversely, during repolarization the transmitter population will recover, provided that there is chemical potential available for the restoration. Note, irrespective of which way the energy gradient lies between the system and its surroundings, free energy can only decrease, and hence entropy can only increase. At the maximum entropy state all forms of free energy have been consumed, and accordingly all energy is bound in the stationary populations. Then there are no net forces that would drive the system away from the thermodynamic balance. Many a living system hardly ever resides in thermodynamic balance because its surroundings keeps changing, but formally Equations (2–6) do express the path-dependent, and hence intractable evolution toward balance as well as complex dynamics at the balance.

It is worth underscoring that entropy by Equation (4) does not convey any additional information about the system than what is given in energetic terms, i.e., by multiplying *S* with *T*. Thus, the least-time free energy consumption means that entropy will not only increase but it will increase at the maximal rate. Importantly, *S* does not relate to disorder, i.e., incoherence. Order or disorder is no end in itself but a mere consequence of free energy consumption. Organization, just like disorder, follows from the quest of consuming free energy. The widespread but unwarranted association of entropy with disorder dates back to the derivation of entropy for closed systems by Boltzmann. Obviously, when the system is defined as invariant in energy, nothing can change per definition. However, life is all about changes, and for that matter, in the expanding Universe no stationary motion will last forever either.

### A neural network as a thermodynamic system

Thermodynamic terms are commonly used in metabolism, but seldom applied in the context of cognition. However, there is no principal difference. Electromagnetic potentials of nerve cells arise from chemical potentials, and hence neuronal signaling can be expressed alike, in terms of the scalar potential *U* = ∫μ*dN* due to bound quanta and in terms of the vector potential *Q* = ∫Δ*QdN* due to absorbed or emitted quanta. Since Equations (1–6) apply also for electromagnetism (Tuisku et al., 2009), a network of neurons engaged in cognition is by thermodynamic principle no different from a reaction network of chemical compounds involved in metabolism. The neural network also evolves, just as the chemical reaction mixture, by consuming free energy in least time (Hartonen and Annila, 2012). For example, evolution of a neural network from one state to another is about accumulating products, e.g., physically embodied representations of experiences and memories. Likewise, lapses and larger losses of memories or mental skills invariably involve changes in the neural network. However, we make no attempt to specify what these changes are in detail, say in diagnostic terms, we only claim that whatever they are, they all are formally contained in the systems theory.

In concord with naturalistic consent we reason that all cognitive processes, for example, learning is ultimately embodied in neuronal systems or in some other systems. The chosen definition of a system is inconsequential because all systems amidst surrounding systems are perceived to evolve, develop and mature, that is, to change from one state to another in one way or another by consuming free energy in least time. Therefore, irrespective of how one chooses to demark the system from its surrounding, the bookkeeping of quanta in the system and of the quantized influxes and effluxes across the interface is perfect.

The freedom for one to define a system does not mean that the classification would be meaningless. Namely, a natural interface is there where strengths of interactions change significantly. For example, neurons in the central nervous system (CNS) are more strongly connected to each other than to rest of the body. Accordingly, the brain and spinal cord are recognized as subsystems of CNS, and, in turn, medulla, pons, thalamus, hypothalamus, cerebellum, hippocampus, basal ganglia, etc., can be recognized as subsystems of brain by their high internal connectivity. The natural interfaces are not impermeable, only fluxes across them are less intense than fluxes within the system.

Connectivity as the natural determinant of a system manifest itself, for instance, when connections across callosum are progressively reduced. The split-brain condition, where the two lobes behave as distinct systems, does not emerge gradually but abruptly (Tononi and Koch, 2015). We claim that the threshold is reached when the free energy consumption via inter-hemisphere connectivity falls significantly below the free energy consumption via the intra-hemisphere connectivity. The underlying principle is the same when two persons grow apart, they will at some stage speak out the split. Likewise, when two populations in a country grow more and more apart from each other, they will at some point declare themselves as two independent nations.

It is worth stressing that the least-time imperative does not specify any particular outcome, e.g., a split or union. This means, for instance, that a memory circuit has evolved to consume free energy by making an “appropriate” recollection, not by recollecting an event exactly as it actually took place. This energetically optimal conduct is customarily referred to as survival. One may easily imagine circumstances where a frank, yet unfaithful recollection will be vital, and scenarios where an exact recollection would be fatal. The same conclusion has been expressed in terms of utility in the context of vision (Purves et al., 2015). Indeed, it is no new thought to think of cognition as a means of survival, but still some might find it unusual to speak about the fittest in thermodynamic terms without making any distinction between animate and inanimate. It is this universality of thermodynamics from which we draw insight to consciousness.

## Consciousness by the thermodynamic tenet

According to thermodynamics there is nothing extraordinary about consciousness; why it exists, what it does and how it arises. On the contrary, its existence, functions and arousal follow from the universal imperative. The Equations (1–6) express in quantitative forms the general biological position that consciousness is a result of evolution, among all other characteristics. Thermodynamically speaking flows of energy will naturally select those characteristic paths that will level off energy differences in least time. According to this perspective, consciousness integrates sensory and other inputs with recollections and representations from the past for coherent responses to consume energy gradients more effectively than by unconscious deeds. In concord with common sense a conscious person acts in a more meaningful way than an unconscious one. The augmented consumption of free energy means enhanced survival. In the following we will examine by the least-time free energy perspective some well-known questions and established stances about consciousness to enlighten its character.

### On the definition

One hand definitions serve to organize diversity of nature. On the other hand a dividing line creates a problem because everything depends on everything else. The border between one category and another is practical but in the end ambiguous when things change from one to the other. Ultimately one quantum of action is enough to make a change from one category to the other. Most notably it is hard to make a clear-cut distinction between living and non-living, although the notions of animate and inanimate themselves are practical. Similarly, it is unclear what exactly is meant by an economy. For example, is a bee hive part of an economic or an ecological system? Similarly, distinction between consciousness and unconsciousness is useful but ambiguous. The scope of awareness, wakefulness and sentience is wide and vague. Also the range of subjectivity and the sense of selfhood are broad and obscure. Capacity to experience and feel varies from one individual to another as well as from one moment to another in an individual.

Consciousness defies categorization precisely because it is functional. The change is the very characteristic of a conscious system. By the same token, a steady state does not display causal relationships, i.e., irreversibility. A mere exchange of quanta without a net flow of energy between the system and its surroundings does not drive the system from one state to another.

Despite these arguments one could perhaps imagine of defining consciousness exactly by taking a snapshot of it. The still frame, however, would not represent any changes, so it would be devoid of the principle characteristic of consciousness. One could eventually think of enclosing the conscious system by a fictitious border, but only in a stationary system quantized trajectories are closed, i.e., bounded. Put differently, evolving and invariant, just as indefinable and definite, are mutually exclusive attributes.

It is no wonder that philosophers since Descartes and Locke have struggled to pin down essential properties of consciousness, because the definition depends on both the content and context of what is deemed as essential, in fact, functional. For example, search for neural, psychological and behavioral correlates is not free from a preset idea of what consciousness is. Physically speaking, the free energy consumption, i.e., functioning is proportional to the changes in energy, not to some absolute and invariant values of energy, i.e., stationarity. Therefore, the search for a set of neural events and structures implies as if consciousness was bounded by a definition rather than being an open operational notion.

It worth emphasizing that not only consciousness but also many other definitions are ambiguous by depending on the subjective choice of key characteristics. For example, the definition of an ecological community depends on what will be listed as its characteristic organisms. Likewise, the definition of a multi-cellular organism is dictated by the list of its cells. The cell, in turn, is defined by its molecules, and so on. The thermodynamic theory claims that definitions are ambiguous when the change is the principal characteristic.

Obviously our account of consciousness by the universal notation of physics encompassing everything reminds of panpsychism, the philosophy that the mind is not only present in humans but in all things (Seager and Allen-Hermanson, 2015). We see this thought to emerge from the correct comprehension that it is impossible to single out anyone evolving system, specifically consciousness, from its surroundings as well as from the accurate observations that all systems behave in the same way, that is, consume free energy in least time. Then again, there is hardly a point in equating the specific notion of mind with the general notion of an evolving system. Thus, we refer to mind merely as a practical term for what the brain does. Likewise, we choose to speak about consciousness merely as an attribute for an integrated system that is consuming free energy coherently.

Still, one might regard consciousness as an umbrella term, for example, in analogy to furniture which, as a term, includes tables, chairs, beds, etc. Since furniture refers to movable objects that support various human activities such as seating and sleeping, one should ask: What functions does consciousness support? Only to realize that the list will remain open. Thus, there is no closed definition for consciousness.

All in all, the trouble in defining consciousness appears to us as contrived. Problems stem from attempts either to single out or to separate consciousness from its surroundings or to attribute consciousness with some unique rather than universal characteristic. The renowned Cartesian dualism appears to us an unfortunate misinterpretation that *res cogitans*, i.e., the realm of thought would mean an immaterial domain and that *res extensa*, i.e., the realm of extension, would mean the domain of material things. Isn't Descartes only naming the system capable of interoception and exteroception as consciousness and referring to the rest as its surroundings so that their interactions convene in the brain? In our mind the purported qualitative distinction between material and immaterial is not his message. Thus, the mind–body problem of how non-physically labeled beliefs, actions and thinking, etc., relate to the physically embodied human being, appears to us utterly artificial.

### On the quantification

Although consciousness defies a closed definition, it is still quantifiable in terms of entropy (Equation 4). The irrevocable increase in entropy *d*_*t*_*S* ≥ 0 (Equation 5) implies somewhat paradoxically the state of consciousness, measured by *S*, can only increase. This is true when consciousness is understood as the attribute of an integrated system that consumes free energy relative to its surroundings, not relative to some absolute invariant reference.

Despite the relativeness of entropy, one may easily imagine in some absolute terms that the degree of consciousness has been increasing over eons when humans have been consuming energy differences relative to their energy-rich surroundings. Consciousness will flourish when supplies are rich and versatile. Conversely, when the surrounding resources narrow down so that the subject faces hunger, sleep deprivation, stress, etc., consciousness will decrease relative to the arbitrary absolute reference. However, the absolute value of entropy, high, or low is only imaginary because entropy is in relation to resources, i.e., a function of free energy (Equation 4). In biological terms the cognitive capacity will adapt to circumstances. In thermodynamic terms the cognitive system will regain balance with its surroundings either by acquiring or abandoning some subsystems and paths of energy transduction. Thus, a high level of consciousness is no end in itself but consciousness, as any other attribute of a system, develops and evolves to attain the entropy maximum, i.e., the free energy minimum state in a given circumstances. This is, of course, common sense. In poverty a high level of awareness is simply unaffordable.

In practice there is hardly a way to sum up numerous bound and free forms of energy to quantify consciousness. Above all it is difficult to gauge all forms of free energy that are represented in one's neural network. These energy differences reside between the system, known as the conscious self, and its surroundings. For one thing, one's perception of its surroundings is dynamic. For the other thing, one's identity, i.e., the system itself is an ambiguous and dynamic notion that prevents from defining it as distinct from its surrounding systems. For example, the problems of altruism and tragedy of commons resolve by identifying one's identity (Annila and Salthe, 2009; Anttila and Annila, 2011).

Although exact quantification of consciousness remains illusory its characteristics can be recognized from the determinants of entropy and its change (Equations 4 and 5). Entropy, as the measure of state, increases with increasing connectivity, not only by an increasing number of nodes, such as neurons, but also by an increasing capacity and rates of mutual interactions (Figure 1). Thus, it is no coincidence that the brain with the fastest processing capacity and highest connectivity among all organs is the primary premise of consciousness. Conversely, the conscious capacity will degrade when connections and central nodes disintegrate but remains largely untroubled by solitary losses. Still, it is worth emphasizing that the comparison in absolute terms of entropy has no real meaning because any state of consciousness is in relation to its resources. High holism remains only imaginary when there are no resources and no means to attain it.

### On the subjectivity

The subjective nature of consciousness is inherent in the thermodynamic account (Figure 1 and Equations 1–6). Namely, the system is the subject. The system is unique via its interactions with its surroundings. A flow of quanta from the surroundings to the system is not shared by any other system. For example, the photon that one's retina happens to absorb cannot be absorbed by anyone else. Accordingly, there is no objective way of defining or measuring any system because any observation will ultimately embody a unique flow of energy from the target to the specific observer. Thermodynamics of open systems acknowledges this uniqueness, i.e., the subjective character of nature. The theory works even when the system is defined at will, because it keeps track of all quanta that move between the system and its surroundings.

All meanings presented in various forms of free energy are subjective. The way things appear to one depend on who one is, that is to say, on evolutionary courses of human species and its ancestors as well as on one's own developmental processes and experiences. Common sensations imply the same origin and ordinary experiences where singular sensations indicate diversification. Since no objective account can be given, it is best to realize consequences of subjectivity. For example, one may begin by defining gamma waves as a necessary, yet insufficient characteristic of consciousness (Aru et al., 2002), and proceed by including other characteristics. When completed with one's list, one may label consciousness as impaired or disrupted when anyone of the predefined characteristics is missing or misplaced.

Neuronal and behavioral correlates of consciousness are undoubtedly needed for medical diagnoses and other purposes, but they are neither comprehensive nor objective. For instance, alcohol and other drugs, or spiritual and meditative techniques will alter the state of consciousness. This is sensed by the subject itself and other subjects, but differently since flows of energy are different. In turn, denial of impairment is a striking example where the subject's view of consciousness is deemed by others as disturbed (Hirstein, 2005). The subjective character of consciousness manifest itself pronouncedly when a patient, who has become blind, claims to see normally and continues to maintain the view despite all evidence to the contrary. This is perplexing, yet ordinary in another context. Isn't it only common that despite all evidence to the contrary, many an individual retains unrealistic thoughts about himself? Also, it is not unusual that one assures of recalling an event which never happened. Consciousness is not and it does not even aim to be a faithful, say objective or inter-subjective representation of reality. It is one's response to reality.

According to thermodynamics a conscious system forms from its constituent systems, like any other integrated hierarchy, The conscious system will consume free energy along the least-time paths, irrespective of how irrational these paths are judged “objectively” by other systems. For example, the changed meaning of a percept demonstrates how a tapered connection will redirect signals, i.e., flows of energy, from a sensory system to an “incorrect” locus at the cortical system. It is odd but still understandable that one may sense the sound of trumpet as “scarlet” (Krohn, 1892). The erroneous outcome is no different from a train arriving on a wrong platform because of a misplaced switch along the track. Put differently, the curious complications are not normal but natural according to the scale-free thermodynamic imperative. Our viewpoint of subjectivity as a natural characteristic complies with monistic consent that consciousness is a real subjective experience embodied by physical processes in the brain. This view is compatible with so-called biological realism at the interface between neural and mental phenomena (Revonsuo, 2006; Freeman, 2007; Fingelkurts et al., 2009, 2010a, 2013).

### On the hierarchy

The scale-free thermodynamic theory pictures the conscious system as comprising of systems (Salthe, 1985; Chialvo et al., 2007; Fingelkurts et al., 2013; Werner, 2013). Consciousness supervenes via least-time energy transduction from lower-level systems, say neuronal networks that represent sensations, coordination, memories, etc. In other words, knowing with oneself integrates existing systems with inputs from surroundings. This is to say, consciousness emerges in a form that best serves the least-time imperative rather than being a comprehensive report of either the state of mind or the state of surroundings. This conclusion about consciousness, as an integrated hierarchal construct, agrees with the impression that consciousness is the opinion or internal feeling that one has from what one does. Also that consciousness is deemed as unitary we understand as the coherent outcome of integration, not that it would mean a monolithic entity.

The view of consciousness supervening lower-level processes parallels the proposition of various narrative fragments, “drafts,” coming together the way a coherent behavior of an individual calls for (Dennett, 1991; Chafe, 1994; Varela, 1999; Freeman, 2007; Fingelkurts et al., 2010a, 2013). The need in thermodynamic terms is a force that will expire by the least-time free energy consumption. In view of that it is natural that new aspects about oneself will surface to one's mind first when one senses corresponding driving forces. As long as one has no mechanisms to sense such forces, it makes no difference if someone else is aware of them. The blind is unaware of her beautiful face, but when learning about it from others, may make all the difference. In general, when sensory outputs from the surroundings are deprived by and large, it will become difficult to maintain a focused state of consciousness. The loss of external energy gradients results in a peculiar state of consciousness where theta waves prevail (Ballard, 1986). These low-frequency oscillations disperse farther away than gamma waves. The extended scale of coherence underlines that consciousness is at its brightest as a focused construct. Yet, consciousness does not reside at any distinct locus in the neuronal network but integrates functional loci to an attentive response (Baars, 1988; Seth et al., 2005; Revonsuo, 2006; Tononi, 2008; Fingelkurts et al., 2010a, 2013; Marchetti, 2012; De Sousa, 2013). Then again, holism is emphasized when an optimal reaction recruits a broad range of processes, including also unconscious functions.

Moreover, consciousness embodying to-and-fro flows of energy (Figure 1) is consistent with observations that activity in primary sensory areas alone is insufficient for consciousness (Koch, 2004). Higher brain areas, especially the prefrontal cortex is involved in a range of cognitive functions, so that executive functions sum up from frontal cortex inputs and also so that neural activity propagates down to sensory areas (Crick and Koch, 2003). These up-and-down flows, so to speak upward and downward causation (Kim, 1984; Meyering, 2003), are consistent with a conscious system resulting from integration systems for the least-time free energy consumption (Figure 1).

### On the hard problem

The so-called hard problem of consciousness is about how a physical process in the brain gives rise to subjective experience (Chalmers, 1995). The eminent claim is that even complete knowledge of the brain would not yield complete knowledge of conscious experience. The assertion means, for example, that even if one knew everything about how the brain processes colors, one would not know what it is like to see them.

According to thermodynamics subjectivity is the characteristic of any system. It is the only option. Subjectivity is not only associated with experience, but equally so with information processing such as reasoning, reporting, focusing attention, etc. Since many immediate stages of information processing at sensory organs are known in quite some detail, it may seem as if there were nothing subjective in the elementary processes, e.g., following the photon absorption at retina. But there are subtle differences among the involved entities. One retinal molecule, as a system of atoms, may seem identical to another one, but each setting is unique. The energy differences, say electromagnetic fields, about the molecule are dictated by everything else, e.g., by other molecules, whose coordination is not identical, i.e., symmetrical for anyone molecule. When the surroundings is unique, also the system is unique, which manifests itself, for instance, as a unique molecular conformation. Undoubtedly it would be very difficult to resolve these subtle differences, e.g., fine structure of electronic orbitals imposed by the surrounding fields. This degree of subjectivity, i.e., energy differences between various retinal molecules, is much smaller than that higher up in hierarchy. Using a powerful microscope there is no difficulty to resolve differences in cells that house those seemingly similar retinal molecules. Unmistakably the cells are subjects. Further up along the line of information processing there are more and more diversity, i.e., energy differences among representations. Therefore, we claim that there is no qualitative difference between the elementary percept of a color that is defined by the photon wavelength and the color-induced subjective experience that is represented uniquely by numerous energy attributes of a neuronal network. The degree of subjectivity is ultimately gauged in energetic terms, and hence the subjective experience does not single out from other phenomena.

The specific experience, i.e., the particular series of changes in one's neural system, depends on one's history. The past processes dictate what paths are available as well as what forms of free energy are at disposal to open up new paths or to close down existing paths to represent the experience. Therefore, what exactly one will experience beyond mere perception of light depends on these diverse assets that one has accumulated during life and inherited from ancestors as well as on forces that are imposed by the surroundings. For example, the experience will be moderated when the visual stimulus is accompanied with sound or sense of touch (Witten and Knudsen, 2005).

No question, the mere perception of color is a simpler and more predictable process than the full experience, simply because changes in energy are smaller and less dispersed at the retinal molecules than those associated with the experience of color at cortical levels. Still, we see no evidence that the two processes would be qualitatively different from each other. Put differently, we cannot see that introspection, as knowing about one's mental life, and phenomenality, as having experience about *something it is like*, would be qualitatively distinct from each other. For the same reason, not all of that what is conscious can be categorized simply as introspective or phenomenal. A finer classification beyond introspection and phenomenality is conceivable (Lycan, 1996), but to us the reductionist approach when missing the integrated character of consciousness, does not seem particularly insightful.

Undoubtedly certain aspects of cognition are more accessible for one to report verbally, reason and to control than others such as experiences of sounds, sensations, emotions, feelings, and others coined as qualia. Nevertheless, we see no line of demarcation between introspection and phenomenality. Isn't it exactly about a fine line between introspection and phenomenality why one admires an artist who is able to portray something one cannot quite picture and spokesman who is able voice something what one cannot quite express?

We do not deny that there are various aspects about consciousness, but their categorization is ambiguous, subjective and circumstantial. For example, there are numerous reports from battlefields when pain is not experienced (Morrison and Bennett, 2006). The subject recognizes the loss of a leg and even reasons its immediate consequences pretty much the same way as his comrades, by shouting “bring a stretcher”. The tense circumstances call for vital activities that suppress experiencing the loss thoroughly. Cognition focuses for survival, that is, for the least-time free energy consumption. Only later, when circumstances allow, the meaning of loss, *something it is like*, will be sensed beyond a verbal account. No words will say it all, because walking is distinct from speaking. No images will expose it all either, because walking is distinct from seeing. For one thing, pain is experienced because touch with the leg is lost. For the other thing, agony is experienced because one's identity is at stake. The leg is an integral part of oneself. Sorrow gauges the loss of one's compromised future possibilities as a disabled. One is in for a major restructuring of neuronal representations of oneself to match the new state of affairs. Yet, all what is experienced due to the loss of a leg is ultimately commensurable in terms of free energy. Loss of a toe as a less devastating experience would entail a smaller revision in one's free energy spectrum.

It is common that music, say a certain melody, will trigger a strong subjective experience, when one associates a whole lot with the piece. Similarly, a familiar scene or a memorable scent might move one from one state of consciousness to another. Curiously, many a scientist has described the moment of a discovery as an elation (Birney, 2013). Apparently mere introspection is not enough to construct the full meaning of a sensational discovery. Only the experience by integrating a whole lot more does do the full justice.

Consciousness as an integrative process is best comprehended in holistic terms. In a sense consciousness amplifies, or more accurately inflates, an elementary sensory signal to an experience by integrating various assets from the past. Also Locke's portrayal of consciousness as *the perception of what passes in a man's own mind* we like to read so that consciousness is an inflated perception. Likewise, the idea that consciousness is about broadcasting information around the brain from one's memory bank (Baars, 1988), we like take to mean that consciousness emerges principally from the existing assets whereas the primary trigger makes only a minor component in the final product. The Latin phrase *conscius sibi*, literally as knowing with oneself, provides yet a complementary perspective on the system of systems by emphasizing that consciousness is about sharing the present impulse with representations of the past.

### On the binding problem

The least-time imperative provides perspective also on how brain creates from sensory inputs coherent perceptual experience. This binding problem (Revonsuo and Newman, 1999; Singer, 2001) comprises both the problem of how the brain segregates elements in an input pattern to discrete entities and the problem of how the brain constructs a phenomenological object from the entities. This formulation parallels the metastability concept (Kelso, 1995, 2012; Bressler and Kelso, 2001; Fingelkurts and Fingelkurts, 2004; Fingelkurts et al., 2009).

It is a common experience when listening to a foreign language, that one has a hard time to distinguish individual words. The separation of words is impaired because one's analyzer is not yet tuned to recognize contrasts, i.e., energy differences among sounds. Undoubtedly one's ear is capable of consuming energy differences in the changes of pitch, but in successive stages of neuronal processing the input fails to recruit one's memory to amplify the unacquainted input to meanings. The unfamiliar input does not trigger further consumption of free energy to produce meanings. Likewise, when facing an unusual view, perhaps after being knocked down all of a sudden, one struggles to discern objects in sight because the familiar reference, as the source of meanings, is tilted. Therefore, we argue that elements in sensory inputs are segregated by consuming free energy in least time. This involves mechanisms that have been established in the course of one's life as well as inherited from the course of evolution. Thus, the outcome of segregation is subjective and context-dependent. One looks for meanings every day, yet practically every textbook of biology shuns the idea that there are meanings and purposes, physically speaking forces, in nature.

Consistently, the construction of an object from the segregated elements is guided by the least-time free energy consumption. The brain is equipped with mechanisms from the past to assemble an object from the segregated ingredients as well as from those ingredients that are available in memory for the integration process. It is not unusual to jump into conclusions which demonstrates that the construction is not and does not even aim to be faithful and consistent. It is biased by prior expectations, physically speaking, by energy gradients. One's conclusion is motivated by gradients in the evolving energy landscape that one's neuronal network is representing.

Admittedly, the thermodynamic tenet clarifies only the principle of how experiences are constructed, not mechanistic details of the processes, for instance, in terms of neuronal correlates. Yet, thermodynamics indicates that familiar stimuli will invoke more rapid, intense and wide-spread responses than unfamiliar stimuli, simply because “familiar” associates with what is already present. Put differently, when a lot of free energy is consumed, things make a lot of sense, and conversely nothing is consumed by non-sense. This may well appear as differentiation of brain states during meaningful stimuli vs. non-meaningful stimuli (Boly et al., 2015).

### On the intractability

It is in place to make few remarks on the integration process itself. The evolutionary equation (Equation 5) reveals that motions consume their driving forces which, in turn, affect the motions, and so on. Mathematically speaking the equation is inseparable, and hence it cannot be solved. Thus, there is no algorithm for consciousness. This point is familiar from the Chinese room argument (Searle, 1980). Consciousness emerges in a non-deterministic manner. Yet, supervenience is not a random, i.e., indeterminate, but a path-dependent process. In other words, one does not know exactly what one will think before thinking and one does not know exactly what one will experience before experiencing. We reason that due to intractability certain neural and behavior responses correlate with consciousness at times while at other times they appear as uncorrelated. Therefore, inability to make precise predictions about cognition are ultimately not due to complexity of the process but due to its path-dependent character.

From the thermodynamic perspective probabilistic inference models, most notably Bayesian models (Knill and Pouget, 2004; Bielza and Larrañaga, 2014) including prior knowledge, mimic natural processes, but the modeled probabilities are not faithful representations of energetics (Equation 3), only parameters. Moreover, the original Markov chain does not carry memory of past events, but only the current state determines the probability distribution of the next state. Even when the future state is modeled to depend on a sequence of the past states (Camproux et al., 1996; Seidemann et al., 1996), the ensuing projection, i.e., a trend does not parallel the actual energy gradient because in reality the force is affected by the motion itself.

### On the intentionality

To be directed toward a goal or thing is an apparent characteristic of consciousness (Evans, 1970). By the free energy perspective an intention means a force, that is, an energy gradient. A conscious mind gazes for various forms of free energy and exploits opportunities to consume them. The intention is fulfilled when associated free energy is fully consumed.

Since these driving forces are sensed by oneself, intentions are subjective. Ambivalent intensions imply that one has difficulties in constructing the resultant force. Also ambiguity about oneself may trouble the process. For example, one might commit a crime intentionally, only to realize later that the act in fact hurts oneself. In other words, one's identity was at the critical moment so narrow that only immediate forces manifested themselves.

In addition to conscious intentions there are also other forms of free energy that one is unconscious about, that is, are not integrated for coherence responses. Subliminal stimuli, e.g., presented as flashes, are too short substrates for the construction of consciousness. Nevertheless, these flows of energy will suffice to prime or bias one for an intended action (Loftus and Klinger, 1992). Thermodynamically speaking, the subliminal stimuli shape the energy landscape of one's mind to channel more readily a more comprehensive flow of energy later. In terms of neuroscience the subliminal stimuli lead to construction of some connections but apparently not enough to pave the full way to consciousness.

The thermodynamic tenet gives also a practical meaning to the philosophical concept of free will (O'Connor, 2014). Free will equates with free energy in one's disposal (Annila and Salthe, 2010b). One may execute at most as much as one has free energy in command. Non-determinism means that when one invests free energy to pursue along a path then some other paths are no longer affordable. This is to say, one is responsible as much as one is in capacity to consume free energy. For example, when in captivity, one is limited to act or even to express oneself, and hence free energy only in metabolic form powers free thinking. When deprived from free energy altogether, one has no choices whatsoever to respond by moving from one state to another, i.e., one is no longer responsive.

### On the rate

Why does the experience of oneself reel at a particular rate, at about one “frame” per 100 ms (Potter et al., 2014)? The coherence calls for synchrony (Singer and Gray, 1995). However, consciousness cannot cohere at the maximal firing rate of individual neurons because inputs must exist before they can integrate to the high-level construct. The common experience of escaping from danger by a reflex reaction demonstrates that more time is consumed in integrating awareness than that it takes for subsystems to act. This emphasizes that the notion of self is not a monolith but a composed union.

The reflex reaction demonstrates also that synchrony, e.g., gamma waves of reticular activating system, alone is insufficient indicator of consciousness. For example, visual information can control behavior without producing a conscious sensation. Fluent functions, i.e., automated sequences remain unconsciously generated until a change away from the ordinary happens. This implies that consciousness is a response, physically speaking a reaction to consume free energy, not in an algorithmic fashion, but in a non-determinate way.

Sleep by displaying a wide range of frequencies gives insight to wakefulness. After a daily dose of high-frequency stimulation the sleep, as a natural process, serves to revise the brain's connectivity spectrum toward a free energy minimum partition. In particular, long wavelengths of deep sleep amend long-range connectivity. Sleeping on a problem, exemplifies the value of balancing neuronal network by adjusting connections, i.e., making new thoughts (Bos et al., 2011). In turn, short wavelengths of sleep, characterized by rapid eye movement (REM), tweak the short-range connectivity, but without the objective of conscious free energy consumption. Therefore, vivid dreams do not necessarily make sense, i.e., cohere. The fact that most muscles are paralyzed during sleep, also implies that sleep, by its broadband iterative choreography, consumes in a path-dependent manner a whole range of imbalances in the brain's connectivity spectrum. In contrast, consciousness, by its high-band coherent activity, consumes various forms of free energy in the subject's spectrum of surroundings.

### On the problem of other minds

The question whether animals and organisms in general are conscious or not, is according to the thermodynamic tenet, like many other queries, troubled by ambiguity in defining consciousness. Nonetheless, it is possible to assess the *degree* of consciousness by the free energy consumption. Of course, one can still imagine a system that is conscious but not responsive, as in a lock-in syndrome (Nordgren et al., 1971), but it is inconceivable that a high-degree consciousness as the integrated response would emerge in the first place via minimal interactions with its surroundings.

The thermodynamic tenet asserts that consciousness is subjective in the spirit of the influential essay (Nagel, 1974) *What is it like to be a bat?* The writing argues that an organism is conscious *if and only if there is something that it is like to be that organism—something it is like for the organism*. A dog can be conscious about those forms of free energy that it can access, say, in the form of food and shelter. Its consciousness manifests itself as behavioral correlates, for example, defending its master. A bacterium is likewise consciousness of its free energy sources, say, in the form of sugar, by displaying chemotaxis as its coherent behavioral correlate. An ion is consciousness about its driving forces, say, in the form of electromagnetic fields by responding to them by motion and deformation.

So, what is it like to be a dog, bat or a bacterium? One may relate to another system as much as one shares the same means and mechanisms to consume the same forms of free energy. Apparently the human being shares with the dog some means of companionship, and hence one experiences collaborative behavior associated consumption of free energy somewhat similar to the dog. For one to know, what it is like to be piloting as a bat does, is possible only as much as one is able to navigate solely by hearing echoes. Still, if one were blind, one would value this skill as much as one is able to benefit from it. For one to know, what it is like to be a bacterium, is possible up to the degree that one shares the same metabolic machinery. Of course for one it means hardly anything to digest few sugar molecules. In this respect it is not much of anything to be a bacterium. Though, something when the intake of sugar leads to the integrated function of chemotaxis. Obviously the question about other minds is not only about how much one shares with diverse animate and inanimate the same machinery of free energy consumption but more relevantly about how much shares with other human beings. Indeed, peer support is highly valuable.

The scale-free thermodynamics recognizes consciousness on other levels of natural hierarchy. For example, awareness of a nation accumulates from numerous activities, such as surveys, polls, and compilation of statistics on various things as well as from foreign sources by diverse means. To compare these activities with those that construct consciousness in a human being is, of course, nothing new. Already Hobbes wrote that *Where two, or more men, know of one and the same fact, they are said to be conscious of it one to another* (Hobbes, 1651). Also the Latin word *conscius* by literally meaning *knowing together* implies the scale-free character of consciousness. Specifically, when we claim that a society is consciousness too, we do not exactly mean Durkheim's collective conscience of beliefs and sentiments among members of a society (Durkheim, 1893) but refer to the natural processes that integrate the society for the coherent free energy consumption. These integrated actions, i.e., culture as a whole (Annila and Salthe, 2010b), can be regarded as meaningful or responsible, i.e., conscious. It is not about *analogy* between a consciousness society and a consciousness individual, it is about *equality* because the theory describes both systems exactly the same way.

The scale-free stance is of a practical value. Namely, it is much easier to observe how the society acquires and integrates information to act in an orchestrated manner and how the society cultivates its identity than it is to obtain data and relate recordings from the human brain to responses and development of the self. For example, certain structures, say, claustrum in the brain and a central hub in the computer network are alike critical for the construction of consciousness and situational picture of the nation (Crick and Koch, 2005). Consciousness is unable to manifest itself when claustrum is disturbed, and similarly the government cannot command when the central hub is out of power. However, not any one vital mechanism is the locus of consciousness. Instead consciousness integrates subsystems to a hierarchal construct in a path-dependent manner. This portrayal resembles the model for spotting meanings in percepts that integrates sensory information to virtual associative networks (Yufik, 1998). The thermodynamic theory bears also a clear resemblance to the integrated information theory of consciousness (Tononi, 2008). The universality in the laws of physics has been recognized earlier to underlie characteristics of consciousness such as criticality, self-organization and emergence (Fingelkurts et al., 2013). All in all, we have not put forward a more acute account on consciousness but merely related the prior comprehension to the profound and universal physical basis.

The universality of thermodynamics implicates also artificial consciousness (Russell and Norvig, 2009). Machine's ability to exhibit intelligent behavior equivalent to or indistinguishable from that of a human being is not an issue, because thermodynamics does not make such a classification. Accordingly, a functionally equivalent but non-conscious organism, i.e., a philosophical zombie (Kirk, 2009) cannot possibly have the same survival advantage, i.e., capacity to consume free energy, as the conscious organism. Consciousness is not an epiphenomenon, but a reaction to forces. From this perspective realization of cognitive robotics is not an algorithmic problem, i.e., not a task of automating versatile and fine motor skills. Consciousness entails embedding evolutionary history and life experience, i.e., a long series of changes, to the machine. Without extensive free energy perspective the machine, just like a human being, will be small-minded. In other words such a creature will not have many processes to integrate for a coherent response. According to Equation (1) it will take time to acquire comprehensive cognitive capacity.

## Conclusions

The least-time free energy consumption is hardly a new perspective on consciousness. Our interpretations and conclusions about consciousness are not original either. The main point is holistic. We regard consciousness, like any other phenomenon, as a manifestation of the natural law. Therefore, the proposed percept is falsifiable, not only by measurements of consciousness, but also by observations on anything else that will disprove the axiomatic basis, namely that quantized actions embody everything. Then again, due to our narrow knowledge and lack of expertize we might have reasoned incorrectly or imprecisely how the mind displays the universal principle in some cases, but such lapses do not jeopardize the theory itself, but call for a revision. Unquestionably our account of consciousness is far from being exhaustive, but hopefully it would be exemplary enough to motivate counterarguments and to provoke discourse.

The notion of information, so central in neuroscience, is conspicuous by its absence. To correct for this shortage we maintain that quanta embody also information in one form or another. Accordingly, information is subject to the universal imperative and its manifestation (Karnani et al., 2009). Specifically, information equates with free energy that is consumed by its receiver. In other words, the meaning of a message is subjective. This definition of information in the tangible terms of physics differs from that given by the abstract information entropy (Shannon, 1948).

Obviously there are other consciousness-associated notions besides information which we have not addressed. Just for curiosity we demonstrate the power of considering everything in terms of quanta by inspecting the association of mass with consciousness that was proposed in the popular thriller *The Lost Symbol* (Brown, 2009). The suggestion makes sense, because any change of state, say from conscious to unconscious, invariably involves either emission of quanta from the system to the surroundings or *vice versa*. The dissipated quanta carry energy *E* ultimately to the vacuum characterized by the squared speed of light *c*^2^. The ensuing change in energy *dE* relates to the change in mass by *dE* = *dmc*^2^. The familiar relationship is pronounced in nuclear reactions, but discernable in chemical reactions, and inferable from gravitational changes.

Finally, one might ask: What a conclusion drawn from the least-time imperative stands out as the most insightful? Reminding of the subjective character of consciousness, it may be that only we find it somewhat surprising that consciousness is mostly generated from archives of mind and comparatively little from momentary inputs. On second thought in this way one will generate integrated responses in least time. This revelation allows us to understand also that it is only natural, not belligerent that an unconventional but profound perception hardly finds any place to take root in an established mind.

## Author contributions

The author confirms being the sole contributor of this work and approved it for publication.

### Conflict of interest statement

The author declares that the research was conducted in the absence of any commercial or financial relationships that could be construed as a potential conflict of interest.
